# Mapping risk of leptospirosis in China using environmental and socioeconomic data

**DOI:** 10.1186/s12879-016-1653-5

**Published:** 2016-07-22

**Authors:** Jian Zhao, Jishan Liao, Xu Huang, Jing Zhao, Yeping Wang, Jinghuan Ren, Xiaoye Wang, Fan Ding

**Affiliations:** Disease Control and Emergency Response Office, Chinese Center for Disease Control and Prevention, Beijing, China; Department of Biological Sciences, University of Notre Dame, Notre Dame, USA

**Keywords:** Leptospirosis, Ecological niche modeling, Logistic regression, Maximum Entropy model, Geographic Information System

## Abstract

**Background:**

Leptospirosis is a water-borne and widespread spirochetal zoonosis caused by pathogenic bacteria called leptospires. Human leptospirosis is an important zoonotic infectious disease with frequent outbreaks in recent years in China. Leptospirosis’s emergence has been linked to many environmental and ecological drivers of disease transmission. In this paper, we identified the environmental and socioeconomic factors associated with leptospirosis in China, and predict potential risk area of leptospirosis using predictive models.

**Methods:**

Leptospirosis incidence data were derived from the database of China’s web-based infectious disease reporting system, a national surveillance network maintained by Chinese Center for Disease Control and Prevention. We built statistical relationship between occurrence of leptospirosis and nine environmental and socioeconomic risk factors using logistic regression model and Maxent model.

**Results:**

Both logistic regression model and Maxent model have high performance in predicting the occurrence of leptospirosis, with AUC value of 0.95 and 0.96, respectively. Annual mean temperature (Bio1) and annual total precipitation (Bio12) are two most important variables governing the geographic distribution of leptospirosis in China. The geographic distributions of areas at risk of leptospirosis predicted from both models show high agreement. The risk areas are located mainly in seven provinces of China: Sichuan Province, Chongqing Municipality, Hunan Province, Jiangxi Province, Guangdong Province, Guangxi Province, and Hainan Province, where surveillance and control programs are urgently needed. Logistic regression model and Maxent model predicted that 403 and 464 counties are at very high risk of leptospirosis, respectively.

**Conclusions:**

Our results highlight the importance of socioeconomic and environmental variables and predictive models in identifying risk areas for leptospirosis in China. The values of Geographic Information System and predictive models were demonstrated for investigating the geographic distribution, estimating socioeconomic and environmental risk factors, and enhancing our understanding of leptospirosis in China.

## Background

Human leptospirosis is a waterborne infectious disease caused by pathogenic bacteria called leptospira. Currently, it is one of the most common widespread spirochetal zoonosis and a growing worldwide public health concern [1]. Leptospirosis can be transmitted directly or indirectly from animals to humans, human-to-human transmission occurs very rarely [2]. In recent years, leptospirosis has gained increased attention because of many outbreaks of leptospirosis around the world [3]. Those outbreaks pose a great burden on health systems and cause significant economic and social disruption [4]. The WHO has identified leptospirosis as a neglected tropical disease of global importance, requiring further research to understand its epidemiology, ecology and the disease burden that it causes around the world [2].

In China, a total of 176,450 cases were confirmed in 29 out of the 34 provinces and municipalities from 1991 to 2010, resulting in an average annual incidence rate of 0.70 cases per 100,000 people [5]. Human leptospirosis, therefore, is still an important zoonotic infectious disease in China because of ongoing frequent outbreaks. However, seldom researches have been done to investigate the risk factors of leptospirosis and the geographic distribution pattern in China, which play a fundamental role in controlling this infectious disease.

The emergence of leptospirosis has been linked to many environmental and ecological drivers of disease transmission [6] such as heavy rainfall, consequent floods, temperature, exposure to animals, etc. The ecology of leptospirosis involves very complex interactions among humans, animal reservoirs, leptospires and their surrounding environments [7]. Risk factors for human infection include occupational exposure, recreational activities, cultural factors and socioeconomic circumstances [8]. Geographic information systems (GIS), remote sensing (RS) and ecological niche modeling have been used for investigating a range of infectious diseases related to environmental and ecological drivers [9–11]. GIS can offer an efficient and practical way to directly visualize the dynamics of infectious disease transmission and identify the geographic distribution and risk factors of epidemic outbreaks [12]. GIS has been used in assessing spatial patterns of relationships between local hydrological factors and human leptospirosis occurrence [3], and identifying risk areas for leptospirosis in American Samoa [6]. These maps are critical for guiding allocation of scarce public health resources.

In this paper, we aim to identify the environmental and socioeconomic factors associated with risk of leptospirosis infection, and predict potential risk areas of leptospirosis in China using ecological niche modeling.

## Methods

### Leptospirosis incidence data

**Fig. 1:**
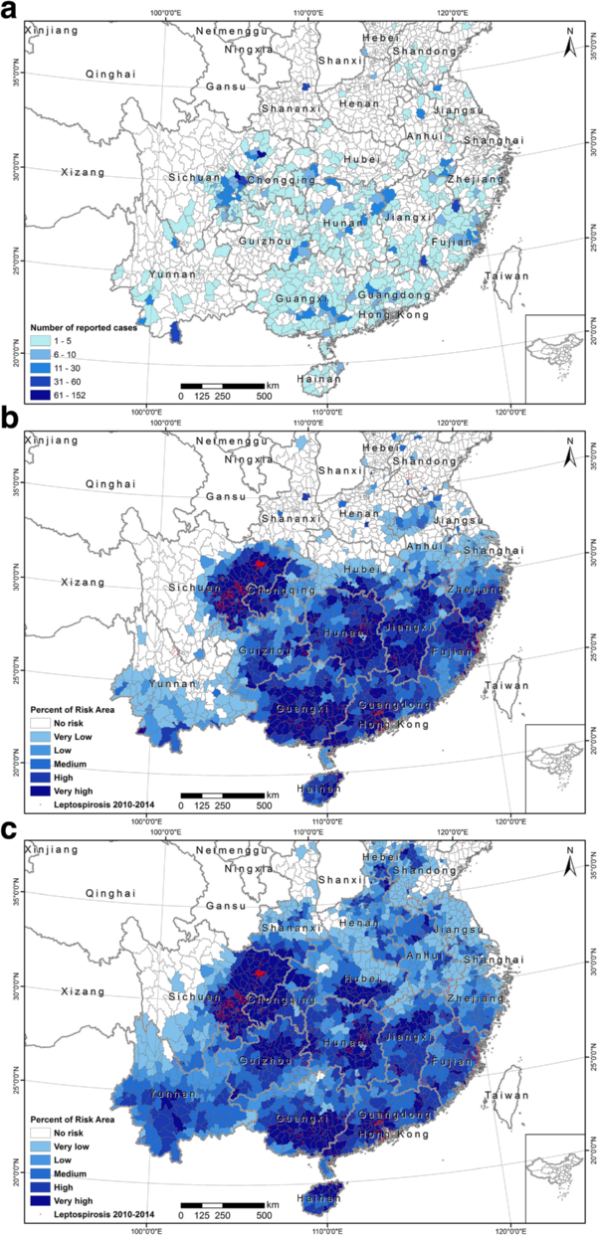
**a** Number of reported cases in each county. **b** Proportion of risk area in each county predicted from Maxent model. **c** Proportion of risk area in each county predicted from logistic regression model

Leptospirosis incidence data were derived from the database of China’s web-based infectious disease reporting system, a national surveillance network maintained by Chinese Center for Disease Control and Prevention (China CDC). Leptospirosis cases were recorded by local hospitals, and disease control and prevention branches of China CDC. Each case was confirmed by laboratory diagnosis tests because the clinical manifestations of leptospirosis were often untypical. According to the surveillance from 2010 to 2014, a total of 2741 leptospirosis cases were geo-coded by using detail addresses or positions of the town. The reported leptospirosis cases are distributed broadly across the south of China (Fig. 1a). After removing duplicated records in the each 1 km × 1 km grid cell (the resolution of environmental data used in this study), we retained 2129 spatially unique occurrence points. We randomly drew 21290 pseudo-absence points from the study area. We combined occurrence and pseudo-absence data to build logistic model and Maxent model.

### Risk factors analysis

Leptospirosis is a multi-factorial infectious disease that is highly associated with both environmental and socioeconomic factors [13].

### Climatic variables

**Fig. 2:**
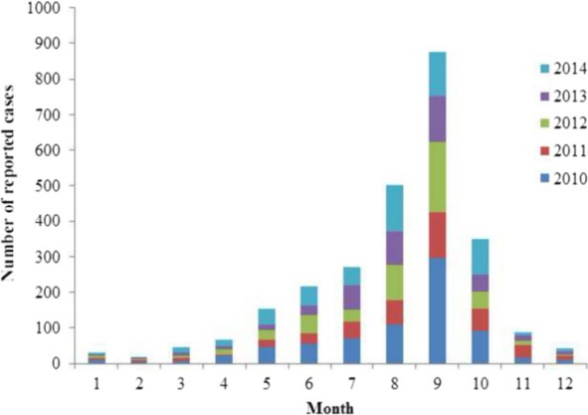
Reported leptospirosis cases from 2010 to 2014 divided by month

Although occurs worldwide, leptospirosis is most prevalent in tropical and subtropical areas with high rainfall [2]. Heavy rainfall events and floods increase the risk of leptospirosis by bringing leptospira and their animal hosts including rat and pig into closer contact with humans [14]. A high correlation between seroprevalence of infection and heavy rainfall and flood events in China was also reported [15]. Leptospires are able to survive for prolonged periods of time in higher temperatures and humid environments [1, 16]. In addition, we found that 37 % cases occurred in summer and 50 % cases in autumn (Fig. 2) in China. Therefore, four variables (annual mean temperature, temperature seasonality, annual precipitation and precipitation seasonality) were obtained from Worldclim database [17], which was interpolated from weather station records from 1950–2000.

### Animal host density variable

Leptospirosis is found mainly in area where humans come into contact with the urine of infected animals or urine- contaminated environments [2]. Rodents and some domestic animals such as pigs and dogs are considered to be the most dangerous for transmitting leptospires to humans [18]. Leptospirosis is considered the only epidemic-prone infection that can be transmitted to humans directly from water, soil and food contaminated urine from infected animal hosts [1]. We considered only the effect of pig density on transmission of leptospirosis. The pig density data was obtained from the Food and Agriculture Organization (FAO) (available at http://www.fao.org/ag/againfo/resources/en/glw/GLW_dens.html).

### Water reservoir variable

Pathogenic leptospires was reported to be associated with the presence of water bodies including streams, lakes and springs. Therefore, areas with high density of water bodies might be at higher risk of leptospirosis occurrence [3]. Average river density was calculated by dividing the total river length in each community by the community area.

### Land cover type

Land cover and land cover change (LUCC) have been related to the incidence of leptospirosis [3, 8, 14]. Land cover type data was derived from GlobCover Land Cover version V2.3 released by European Space Agency (available at http://www.esa.int/esaEO/SEMXB7TTGOF_index_0.html).

### Economic variable

Leptospirosis cases have been reported in a variety of settings ranging from large urban centers after heavy rainfall events to remote rural areas with limited access to clean drinking water. Leptospirosis is often considered a disease of poverty in middle and low income countries because it affects mostly vulnerable populations [19]. Gross Domestic Product (GDP) is a measurement of the economic performance and living standard. GDP data at county level was obtained from National Bureau of Statistics. Human population density could serve as proxy for the exposure to infected livestock. Gridded human population density data was obtained from Data Sharing Infrastructure of Earth System Science (available at http://www.geodata.cn/Portal/metadata/viewMetadata.jsp?id=100101-38).

### Population density

National 1 km*1 km gridded population density data of 2003 in China was obtained from Data Sharing Infrastructure of Earth System Science (available at http://www.geodata.cn/Portal/metadata/viewMetadata.jsp?id=100101-38).

### Data processing

All data layers were projected in Albers coordinates and resampled to spatial resolution of ~1 × 1 km in ArcGIS Desktop 9.3 (Environmental Systems Research Institute, Redlands, CA, USA) environment. We log-transformed three variables including pig density, GDP and human population density. The final set of predictors is listed in Table 1. We calculated correlation matrix for all variable. The highest correlation coefficient is between log-transformations of pig density and human population density (0.635) (Table 2). Therefore, there is low multicollinearity among predictors.

**Table 1:** Environmental and socioeconomic variables

Risk factors		Data description	Type	Percent contribution	Permutation importance
Environmental factors	Bio1	Annual Mean Temperature	Continuous	47.9	43.5
	Bio4	Temperature Seasonality (standard deviation *100)	Continuous	3.7	5.2
	Bio12	Annual Precipitation	Continuous	34.5	37.8
	Bio15	Precipitation Seasonality (Coefficient of Variation)	Continuous	0.5	1.5
	River Density	Average River Density	Continuous	1.3	2.3
	Log_Pig	Distribution of pig density from FAO	Continuous	1.6	0
Socioeconomic factors	Landcover	Land cover type GlobCover	Categorical	1.7	1.7
	Log_GDP	Gross Domestic Product of county	Continuous	0.7	3.2
	Log_CNPOP	Gridded human population density	Continuous	8	4.7

**Table 2:** Correlation matrix of risk factors

	Bio1	Bio4	Bio12	Bio15	River Density	Log_Pig	Log_GDP	Log_CNPOP
Bio1		−0.3073	0.5914	−0.5531	0.3345	0.5974	0.3559	0.5918
Bio4			−0.3364	0.3409	−0.0174	−0.0838	0.2491	−0.1070
Bio12				−0.4608	0.4835	0.5304	0.2342	0.4596
Bio15					−0.4035	−0.2286	−0.0840	−0.2603
River Density						0.5345	0.3201	0.5067
Log_Pig							0.4422	0.6350
Log_GDP								0.3562
Log_CNPOP								

### Ecological niche modeling

Ecological niche modeling (ENM) has been proved to be an effective tool to investigate the geography and ecology of disease transmission [6, 10]. A logistic regression model and Maxent model were used in this study to predict the potential risk of leptospirosis distribution in China.

### Logistic regression model

Logistic regression is often used to determine the probability of a binary dependent variable based on one or several independent variables. The binary dependent variable represents presence or absence of leptospirosis points. The independent variables are a set of environmental and socioeconomic factors thought to determine distribution of leptospirosis. The advantage of logistic regression is that, through the addition of a suitable link function to the usual linear regression model, the variables may be either continuous or discrete, or any combination of both types [20]. Logistic regression analysis is explained as a linear equation given below.

1 $$ \mathsf{Y}=\mathsf{Logit}\left(\mathsf{p}\right)=\mathsf{In}\left(\frac{\mathsf{p}}{\mathsf{1}\hbox{-} \mathsf{p}}\right) $$

2 $$ \mathsf{Y}={\upbeta}_{\mathsf{0}}+{\upbeta}_{\mathsf{1}}{\upchi}_{\mathsf{1}}+{\upbeta}_{\mathsf{2}}{\upchi}_{\mathsf{2}}+\cdots +{\upbeta}_{\mathsf{n}}{\upchi}_{\mathsf{n}} $$

Where p is the probability that the dependent variable (Y) is 1, p/(1 − p) is the so-called odd or frequency ratio, ${\upbeta}_{\mathsf{0}}$ is the intercept, and ${\upbeta}_{\mathsf{1}}$, ${\upbeta}_{\mathsf{2}}$,…, ${\upbeta}_{\mathsf{n}}$ are coefficients, which measure the contribution of the independent variables (X1, X2,…, Xn) to the variations in Y.

The probability of leptospirosis occurrence can be calculated from 9 environmental and socioeconomic factors (Table 1) using the logistic regression equation and coefficients obtained in R.

### Maxent model

Maximum entropy (Maxent) model [21] is a very popular ecological niche model, which outputs the maximum entropy distribution that satisfies the constraints determined by a set of environmental variables. Maxent uses presence and background points (pseudo-absence points) randomly drew in study area to estimate probability of distribution of leptospirosis. 75 % occurrence points (1597) were randomly selected for training model, and the reaming 25 % points (532) were used for assessing model performance. A total of 21290 pseudo-absence points were randomly sampled from provinces where no case were reported in the period of 2010 to 2014. Nine environmental and socio-economic variables (Table 1) were used to build models to predict the geographic distribution of leptospirosis in China. To measure the relative importance of each environmental and socio-economic variable in Maxent model, a jackknife manipulation was performed.

### Model assessment

Receiver operating characteristic (ROC) analysis was used to evaluate the discrimination ability of the two models, and to determine the optimal threshold to convert probability of distribution of leptospirosis to binary outputs (presence and absence of leptospirosis). The threshold-independent indicator, AUC (area under the Receiver Operating Characteristic curve), was used to evaluate model performance. AUC greater than 0.7 indicates an adequate predictive ability of the model [22]. The optimal threshold that equals test sensitivity and specificity, which gives equal weight to sensitivity and specificity, was selected to identify risk areas of leptospirosis in China. The probability of leptospirosis presence could be converted into presence and absence using this threshold. A set of threshold-dependent assessment indices including sensitivity, specificity, Kappa, true skill statistic, were calculated.

## Results

### Model assessment

Both logistic regression model and Maxent model have high performance in predicting the occurrence of leptospirosis in China, with AUC value of 0.95 and 0.96, respectively. The optimal threshold that equals sensitivity and specificity is 0.05 for logistic regression model and 0.34 for Maxent model, respectively. Five threshold-dependent model assessment indices are shown in Table 3. Our results show that Maxent model performs better than logistic regression model regarding all model assessment indices.

**Table 3:** Model assessment indices

Model performance index	Logistic regression model	Maxent model
AUC	0.95	0.96
Threshold	0.05	0.34
Sensitivity	0.98	0.99
Specificity	0.86	0.89
Proportion of correct prediction	0.90	0.93
True skill statistic	0.84	0.88
Kappa	0.81	0.86

### Main risk factors of leptospirosis

**Fig. 3:**
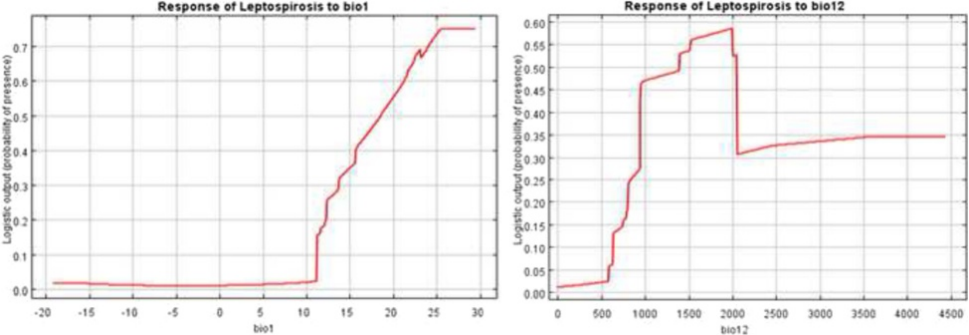
Response curves of two most important variables: bio1 (annual mean temperature) and bio12 (annual total precipitation)

Annual mean temperature (Bio1) and annual total precipitation (Bio12) are two most important variables governing the geographic distribution of leptospirosis in China, with permutation importance of 43.5 % and 37.8 % from Maxent model, respectively (Table 1). They produced the best predictions when used alone from Jackknife analysis. The response curves of the two most important variables reveal that the transmissions of leptospirosis mainly happen in hot and relatively wet regions in China. The probability of leptospirosis presence increases as annual mean temperature increases from 12 °C to 30 °C (Fig. 3). The risk of leptospirosis outbreak increases significantly as annual precipitation increases from 1000 mm to 2000 mm. Compared with the linear relationship between response variable and predictors in logistic regression model, response curve from Maxent model showed that Maxent can build nonlinear and complex relationships between probability of occurrence of leptospirosis and predictors.

### Leptospirosis risk mapping

**Fig. 4:**
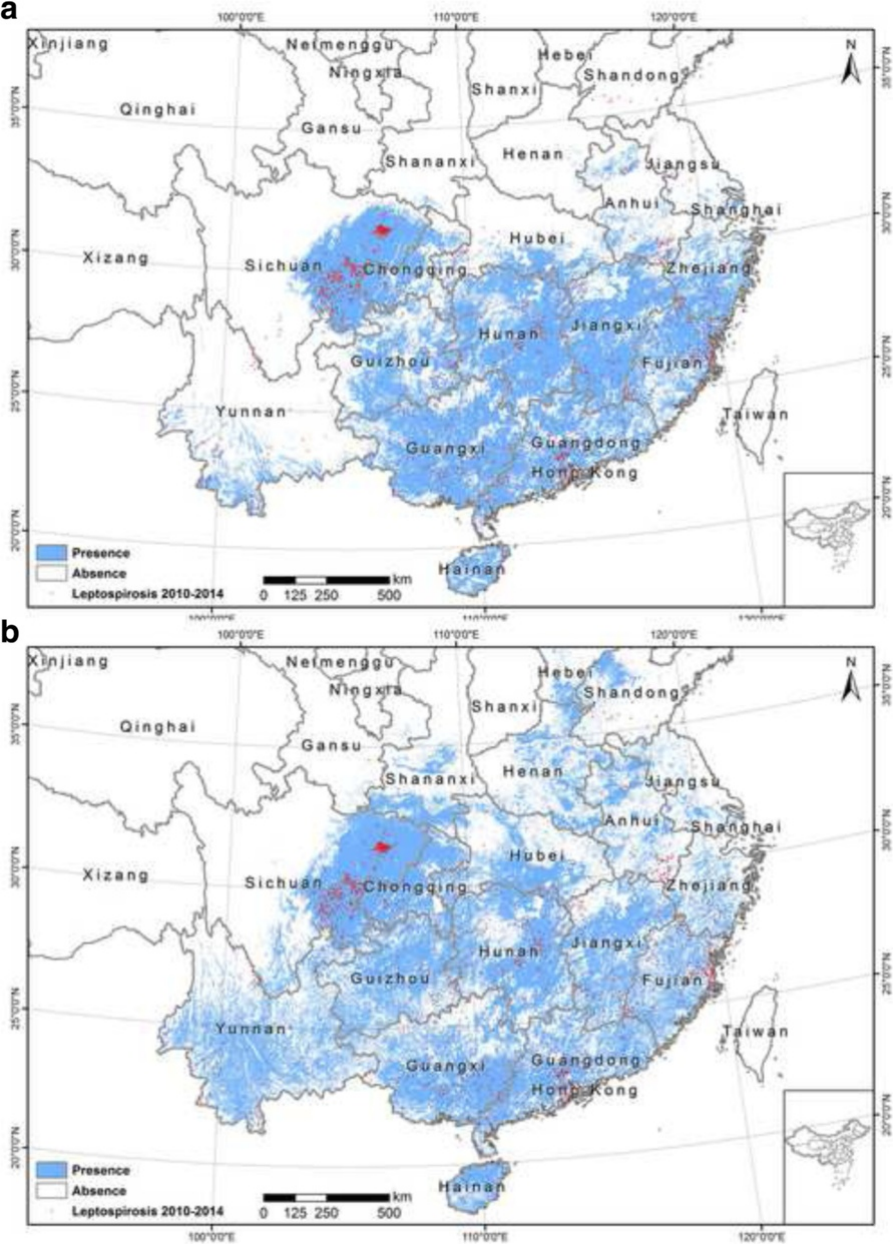
Risk map of leptospirosis in China. **a** Area at risk of leptospirosis in China predicted from Maxent model. **b** Area at risk of leptospirosis in China predicted from logistic regression model

**Fig. 5:**
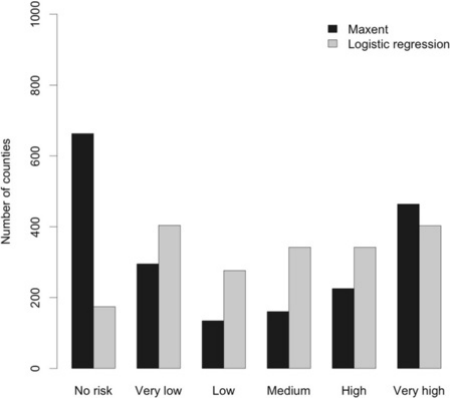
Distribution of leptospirosis risk at county level in China

We converted the predicted maps of probability of leptospirosis presence from the two models to presence and absence using the corresponding optimal thresholds. The areas predicted to be present with leptospirosis are considered as risk area of leptospirosis. Our results revealed that there is high spatial variability of leptospirosis seroprevalence in China. The risk areas are located mainly in seven provinces of China: Sichuan Province, Chongqing Municipality, Hunan Province, Jiangxi Province, Guangdong Province, Guangxi Province, and Hainan Province, where surveillance and control programs are urgently needed (Fig. 4). There is high agreement between the prediction maps from logistic regression model and Maxent model. But the logistic regression model predicted larger risk areas than Maxent model, especially in Yunnan Province. We also calculated the proportion of risk area in each county of all provinces which have reported cases in the period of 2010–2014. Based on the proportion of risk area, we classified all counties into six risk categories: no risk (risk area = 0), very low (0 < risk area < = 0.2), low (0.2 < risk area < = 0.4), medium (0.4 < risk area < = 0.6), high (0.6 < risk area < = 0.8), very high (0.8 < risk area < = 1.0) (Fig. 5). The risk maps show very apparent spatial clustering of counties at very high risk of leptospirosis in China. There are 403 and 464 counties in very high risk category from logistic regression model and Maxent, respectively (Fig. 5). In those counties, disease survey and monitoring program are urgently needed, especially from August to October when most leptospirosis infections happen.

## Discussion

In this study, we compiled surveillance data of leptospirosis in China from 2010 to 2014, and collected a number of environmental and socioeconomic variables thought to determine the geographic distribution of leptospirosis. We used ecological niche modeling approach to better understand the relationship between occurrence of leptospirosis and environmental and socioeconomic variables, and predicted the geographic distribution of leptospirosis in China. Because the reliable absence data are hardly available, random selected pseudo-absence points in provinces having no reported case was used in logistic regression model. According to the model performance assessment, both model can predict the geographic distribution of leptospirosis in China very successfully. Maxent model has better performance compared with logistic regression model, because it can handle non-linear relationships between response variable and predictors.

Pervious studies of epidemiology of leptospirosis have highlighted the importance of socioeconomic and environmental drivers [13]. Environmental variables play a significant role in natural-focal diseases by affecting pathogens directly, or influencing the distribution and abundance of disease hosts and vectors [23]. Four biological meaningful climatic variables including annual mean temperature (Bio1), annual precipitation (Bio12), temperature seasonality and precipitation seasonality, which represent the annual trend and seasonality of climatic condition of leptospirosis transmission, were selected to build predictive models for leptospirosis occurrence. Annual mean temperature (Bio1) and annual precipitation (Bio12) are the two most important factors determining the geographic distribution of leptospirosis in China. Because leptospirosis is often considered as a water-borne infectious disease, we used average river density to quantify the impacts of water condition on leptospirosis transmission indirectly. However, river density variable has no significant effect on predicting the geographic distribution of leptospirosis, possibly due to the coarse spatial resolution variables and the river density is high across the south of China.

Leptospirosis is highly correlated with socioeconomic factors. Leptospirosis occurrence in developing countries is related with intense and rapid urbanization without adequate infrastructure, resulting in sanitation problems, especially in poor vulnerable areas (i.e., slums) located close to rivers or channels, prone to periodical flooding. GDP was a meaningful variable explaining the relationship between leptospirosis and local economic condition. But the GDP data in China was calculated at county level, not at fine spatial resolution of 1 km * 1 km like other variables used in this study. The large number of pigs and backyard piggeries have previously been implicated in leptospirosis transmission [6]. However, we found that no socioeconomic variable was selected as the most important variable in determining the geographic distribution of leptospirosis in China. Other sanitation related variables and population socioeconomic status variables might preform better than the socioeconomic variables used in this study, but those kind of data are not available at fine spatial resolution, and especially at national scale.

Along with the unprecedented global climate change [24], the geographic distribution of leptospirosis would be changed, which need further study. A study revealed that climate change likely contributed to an increase in rodent populations, and therefore the number of outbreaks of leptospirosis [25]. Climate change may increase the incidence rate of leptospirosis infections in the current transmission area and cause the leptospirosis spread to new area, especially in developing countries. Surveillance program and control program is urgent needed in the counties identified as high risk county or very high risk county.

A comprehensive approach involving humans, animals and the environment is key to developing targeted control program [26]. The China CDC and its branches are officially required to report monthly and yearly information on leptospirosis, including the incidence, mortality and major animal hosts, to the Ministry of Health of China. We successfully identified the most influential risk factors of transmission of leptospirosis, and the risk level of counties in China. Our results could help to improve the surveillance program and control program of leptospirosis in China. The current available prevention and control program include improving in sanitation and living conditions, reducing the leptospirosis infection rate in animal hosts, and targeting vaccinations to high risk populations. Our risk maps of leptospirosis could help to optimize the allocation of public health resources, particularly in areas with limited financial and public health resources.

## Conclusion

Ecological niche modeling approach is a great tool to enhance our understanding of the drivers of leptospirosis transmission at large scale, and predict the geographic distribution of leptospirosis in China. Our risk mapping results highlighted the importance of socioeconomic and environmental variables in identify risk areas for leptospirosis. Future study should be aimed at including more meaningful risk factors and using spatial-temporal modeling to investigate the seasonal and interannual variability of leptospirosis risk in China.

## Abbreviations

AUC, area under the receiver operating characteristic curve; Bio1, annual mean temperature; Bio12, annual precipitation; Bio15, precipitation seasonality (Coefficient of Variation); Bio4, temperature seasonality (standard deviation *100); China CDC, Chinese Center for Disease Control and Prevention; ENM, ecological niche modeling; FAO, Food and Agriculture Organization; GDP, gross domestic product; GIS, geographic information system; Landcover, land cover type globcover; Log_CNPOP, gridded human population density; Log_GDP, gross domestic product of county; Log_Pig, distribution of pig density from FAO; LUCC, land cover and land cover change; Maxent, maximum entropy model; River Density, average river density; ROC, receiver operating characteristic ; RS, remote sensing; WHO, World Health Organization
